# Clinical Significance of Respiratory Involvement in Cryptosporidiosis: Cross-Sectional Study of Children with Diarrhea and Respiratory Symptoms in Uganda

**DOI:** 10.4269/ajtmh.24-0112

**Published:** 2024-08-13

**Authors:** Siobhan M. Mor, Grace Ndeezi, Luke R. Ascolillo, Hannington B. Tasimwa, Charalampos Attipa, Jerlyn Sponseller, David Mukunya, Ritah Nakato, Lilian N. Kayondo, Saul Tzipori, James K. Tumwine, Jeffrey K. Griffiths

**Affiliations:** ^1^ Institute for Infection, Veterinary and Ecological Sciences, University of Liverpool, Liverpool, United Kingdom;; ^2^ Department of Paediatrics and Child Health, School of Medicine, Makerere University College of Health Sciences, Kampala, Uganda;; ^3^ Department of Public Health and Community Medicine, Tufts University School of Medicine, Boston, Massachusetts;; ^4^ Department of Microbiology, Makerere University College of Health Sciences, Kampala, Uganda;; ^5^ Department of Infectious Disease and Global Health, Tufts University Cummings School of Veterinary Medicine, North Grafton, Massachusetts;; ^6^ Department of Community and Public Health, Busitema University, Busitema, Uganda;; ^7^ Department of Paediatrics and Child Health, School of Medicine, Kabale University, Kabale, Uganda;; ^8^ Department of Civil and Environmental Engineering, Tufts University School of Engineering, Medford, Massachusetts;; ^9^ Tufts University Friedman School of Nutrition Science and Policy, Boston, Massachusetts

## Abstract

Respiratory cryptosporidiosis is considered an occasional, late-stage complication of HIV/AIDS. This study aimed to assess the clinical importance of respiratory cryptosporidiosis in children with diarrhea and respiratory symptoms at Mulago Hospital, Kampala, Uganda. Children aged 9 to 36 months presenting with diarrhea and cough or unexplained tachypnea (*N* = 1,918) were screened for fecal *Cryptosporidium* using polymerase chain reaction (PCR). Children with positive stool samples were eligible for further diagnostic tests, including sputum induction. Sputum samples were subjected to PCR for *Cryptosporidium*, as well as routine microbiology (culture and gram stain) and auramine stain for tuberculosis. Regression analyses were used to investigate 1) factors associated with respiratory cryptosporidiosis and 2) whether respiratory cryptosporidiosis was independently associated with hospitalization. Prevalence of enteric cryptosporidiosis was 260/1,918 (13.6%) (>80% *Cryptosporidium hominis*). Of the 236 children who had sputum available for analysis, 62 (26.3%) had *Cryptosporidium* in the sputum, only two of whom had HIV infection. Children with *Cryptosporidium* in the sputum were more likely to have abnormal oxygen saturation at presentation (SpO_2_ <96%; *P* = 0.053); no other differences in frequency or severity of respiratory signs were noted. No alternative bacterial cause of respiratory symptoms was identified in 37.7% of children with respiratory cryptosporidiosis, compared with 23.6% of children without (*P* = 0.04). Sputum-positive children had twice the odds of hospitalization compared with children without *Cryptosporidium* infection at this site (adjusted odds ratio = 2.08, 95% confidence interval: 1.02–4.22; *P* = 0.043). Respiratory tract involvement is common in children with intestinal cryptosporidiosis who are experiencing respiratory symptoms. Such children may experience some degree of respiratory compromise and may be at increased risk for hospitalization.

## INTRODUCTION

*Cryptosporidium* spp. is estimated to be responsible for nearly 50,000 diarrheal-associated deaths in pediatric patients annually and contributes to more than 12 million disability-adjusted life-years lost globally.^1^ Several multicenter studies have confirmed the protozoan as a major cause of diarrhea in children under age 2 years.^2^^–^^6^ The parasite has a worldwide distribution, with *Cryptosporidium hominis* (largely anthroponotic) and *Cryptosporidium parvum* accounting for most human cases.^7^ Although the latter is often considered zoonotic, sequencing revealed that the majority of *C. parvum* genotypes recovered in the Global Enteric Multicenter Study (GEMS) study were in fact anthroponotic.^8^ Despite the screening of hundreds of agents, no therapeutic agent has been found to be consistently effective against the parasite. Nitazoxanide has shown partial efficacy against *Cryptosporidium*^9^ and is licensed for use in some countries; however, it is not effective in immunocompromised patients. Although its use is increasing, nitazoxanide remains unavailable in most developing countries. No vaccine is available.

Cryptosporidiosis is typically a self-limiting illness (<14-day duration) with clinical infection localized to the gastrointestinal tract in healthy individuals. Watery diarrhea is the typical symptom in children, with many experiencing subsequent growth faltering.^10^ Infection can become life-threatening in people who are immunocompromised, such as those with untreated HIV infection and malnourished children aged 6 to18 months, due to prolonged diarrhea (≥14 days), weight loss, and associated severe dehydration.^8^ In the GEMS, *Cryptosporidium* was one of only a handful of pathogens associated with increased risk of death in children with moderate-to-severe diarrhea under age 2 years.^3^ Respiratory tract infection has also been widely documented,^11^^–^^19^ although this presentation is typically recognized as an end-stage complication of HIV infection (see the comprehensive review by Sponseller et al.^20^). Several studies in the general population have documented a high frequency of respiratory tract symptoms in children and adults with intestinal cryptosporidiosis,^21^^–^^25^ leading to the hypothesis that respiratory involvement may be more common than is currently recognized.

We previously reported that 35% (17/48) of stool-positive children presenting with diarrhea and cough to the Acute Care Unit (ACU) of Mulago Hospital, Uganda, had *Cryptosporidium* in the sputum.^19^ Here we present the findings from a subsequent study designed to assess the clinical significance of respiratory tract cryptosporidiosis in the same population. We hypothesized that respiratory tract infection with *Cryptosporidium* would be independently associated with respiratory compromise and poor outcomes, the prevalence and magnitude of which may be modulated by factors such as HIV infection, malnutrition, and concurrent respiratory pathogens.

## MATERIALS AND METHODS

### Study population.

Male and female children aged 9 to 36 months who presented with diarrhea or cough as the chief complaint to the ACU of the pediatric wards of Mulago Hospital in Kampala, Uganda, were screened for eligibility. The younger end of the age range was selected based on the ability of children to cooperate with study procedures and the older end of the age range was based on the high prevalence of cryptosporidiosis in this age group.^26^^,^^27^ Children were eligible if they had (acute or persistent) diarrhea and cough or unexplained tachypnea. Diarrhea was defined as ≥3 loose stool in the previous 24 hours or any number of bloody stools in the previous 24 hours (≤14 days duration for acute diarrhea; >14 days duration for persistent diarrhea). Children of unknown age, as well as those who had known medical conditions (cardiac, central nervous system, metabolic, or endocrine disorders), recent history of choking, suspected foreign body inhalation, who were clinically unstable, or who were moribund were excluded.

### Clinical procedures.

All children underwent basic clinical evaluation including assessment of respiratory rate and pattern, chest auscultation, cutaneous oxygen saturation (using pulse oximetry), and nutritional status (height/length, weight, mid-upper-arm circumference and head circumference). Respiratory rate was assessed after rehydration in children who were dehydrated. Oxygen therapy was initiated in children with hypoxia (oxygen saturation [SpO_2_] <92% on admission). Stool samples were collected as soon as children were in a stable condition, which was typically achieved within 2 hours of presentation at the ACU. Stool was screened for the presence of *Cryptosporidium* DNA using polymerase chain reaction (PCR; discussed subsequently). All stool-negative children exited the study at this stage, whereas all stool-positive children were subsequently screened for eligibility for sputum induction (discussed subsequently). Children were hospitalized or not based on the independent clinical judgment of the ACU clinical staff without regard to their *Cryptosporidium* status.

Blood was obtained by venipuncture in children being considered for sputum induction. Children were eligible for sputum induction if they had unexplained cough, tachypnea, or hypoxia per the preceding definitions. Children were excluded from the procedure if they had asthma, chronic lung disease (including any suppurative lung condition), known hypersensitivity to salbutamol or cautions to its use (convulsive disorders, glaucoma, and sensitivity to sympathomimetic amines), hypoxia refractory to 30 minutes nasal oxygen therapy, thrombocytopenia (platelets <50 × 10^6^/mL), or hypokalemia (potassium <3.5 mEq/L).

Sputum induction was performed in eligible children according to the Mulago Hospital guidelines. Before the procedure, saliva was collected from each child using a Versi-SAL oral fluid collection device (Oasis Diagnostics, Vancouver, Canada). Children were then pretreated with salbutamol administered via an inhaler or nebulizer (0.1 mg/kg in 5 mL of sterile saline). This was immediately followed by administration of 5 mL of 3% hypertonic saline using an ultrasonic nebulizer for 5 minutes to induce sputum. Sputum was obtained by suctioning through the nasopharynx with a sterile size 6 nasogastric tube attached to a 10-mL syringe. Children were monitored throughout the entire procedure (including pulse oximetry) and for 4 hours after the procedure before discharge.

### Laboratory analysis.

DNA was extracted from stool, sputum, and saliva using QIAamp first DNA stool mini kit and QIAamp DNA mini kit for sputum and saliva (Qiagen, Hilden, Germany) according to manufacturer’s instructions. Samples were tested for *Cryptosporidium* spp. at the research laboratories of Makerere University College of Health Sciences using a two-stage testing protocol. Briefly, after DNA extraction, specimens were screened for *Cryptosporidium* spp. using a SYBR-based, nested PCR assay targeting the 18 s rRNA gene. Samples with a positive determination (Ct ≤33) were subjected to a further round of real-time PCR using species-specific probes designed to distinguish *C. hominis* (small subunit rRNA gene), *C. parvum* (SSU rRNA gene), and *Cryptosporidium meleagridis* (60-kDa glycoprotein 60). Reaction volumes and thermocycler conditions are provided in detail in Mor et al.^17^ Sputum was also submitted to Mulago laboratories for routine bacteriology (Gram stain and culture) and tuberculosis testing (auramine stain). Where sputum volume was insufficient, diagnostic testing for pathogens was prioritized over research testing.

Blood was submitted to Mulago laboratories for complete blood count (including hemoglobin and platelets), albumin, electrolytes, and routine HIV testing. Electrolyte levels were assessed using the photometric method (COBAS 6000, c 501 module automated clinical chemistry analyzer). HIV testing was conducted using standard procedures at Mulago—namely, serial ELISA with confirmatory HIV RNA PCR in children aged <18 months.

## STATISTICAL ANALYSES

The primary parasitological outcomes of interest were presence of *Cryptosporidium* spp. in stool, sputum, and saliva. Primary clinical outcomes of interest were presence of tachypnea at presentation or, if dehydrated, after rehydration; oxygen saturation at presentation (%, as measured by pulse oximetry); and unfavorable outcome (hospitalization and/or death). Other variables of interest were HIV status; nutritional status (as measured by height/length and weight-for-age); and concurrent respiratory infection. Continuous variables were described using means (SD) or medians (IQR) and compared using *t*-tests or Mann–Whitney *U* tests as appropriate. Categorical variables were described as proportions and compared using χ^2^ tests and Fisher’s exact tests as appropriate.

Logistic regression analyses were used to investigate factors associated with respiratory cryptosporidiosis (model 1) and whether respiratory cryptosporidiosis was associated with hospitalization (model 2). Analysis was limited to those stool-positive children who underwent sputum testing for *Cryptosporidium*. Independent variables of interest in model 1 were history of vomiting and presence of *C. hominis* in stool. For model 2, the independent variable of interest was presence of *Cryptosporidium* in sputum. Gender, age, HIV status and nutritional status were included as confounders in both models. Additionally, hydration status was included as a confounder in model 2. Data analysis was performed in IBM SPSS Statistics for Macintosh (version 29; IBM Corp., Armonk, NY), and plots were prepared using the ggplot2 package in R Statistical Software (version 4.1.2; R Core Team, Vienna, Austria).

### Ethical considerations.

The study was approved by the research ethics committees of Makerere University School of Medicine (protocol no. 2013-001), Mulago Hospital (no. 449), and Tufts Medical Center/Tufts University Health Sciences Campus (no. 10699). Research clearance was also obtained from the Uganda National Council for Science and Technology. Caregivers provided written informed consent in English or the local language (Luganda); caregivers who could not or would not read the information statement provided their consent with a thumbprint after verbal discussion of the document.

## RESULTS

### Participants.

Between May 8, 2015 and January 11, 2017, 1,918 children were enrolled in the study. The demographic and clinical characteristics of enrolled children are shown in Table 1. Nearly 90% of the children were aged under 2 years. Although all children had diarrhea as a matter of eligibility and the majority (63%) had a recent history of vomiting, most (85%) were not dehydrated at the time of recruitment. HIV infection was rare, and malnutrition was present in only 25% of children. Around one-third (35.3%) of enrolled children were hospitalized during the study. Six children died for reasons unrelated to study procedures. All six children who died had underlying severe acute malnutrition and died on the nutrition ward; two of the six children were HIV infected and were naive to highly active antiretroviral therapy.

**Table 1:** Demographic and clinical characteristics of enrolled children aged 9 to 36 months presenting with diarrhea and cough or unexplained tachypnea to the Acute Care Unit of Mulago Hospital, Kampala, Uganda (*N* = 1,918).

Characteristic	Value
Demographic characteristics	
Age, months, median (IQR)	14 (7)
Age category, months	
9–12	791 (41.2)
13–24	945 (49.3)
25–36	182 (9.5)
Male sex	1,086 (56.6)
Respiratory and gastrointestinal signs and symptoms	
Cough	1,911 (99.6)
Diarrhea	1,918 (100)
History of persistent diarrhea*	180 (9.4)
History of recurrent diarrhea*	926 (48.3)
Recent history of vomiting	1,204 (62.8)
Other clinical characteristics	
Hydration status	
No dehydration	1,631 (85.0)
Some dehydration	271 (14.1)
Severe dehydration	16 (0.8)
Nutritional status	
Underweight*	488 (25.6)
Stunted*	388 (20.4)
Wasted*	446 (23.9)
HIV-positive^†^	6 (2.6)
Clinical end point	
Admitted to hospital	678 (35.3)
Length of hospitalization, days, median (IQR)*	3 (3)
Death	6 (0.3)

IQR = interquartile range. Values are *n* (%) unless otherwise stated.

* Data not available for some children: persistent diarrhea (*n* = 1), recurrent diarrhea (*n* = 1), underweight (*n* = 9), stunted (*n* = 18), wasted (*n* = 53), and length of hospitalization (*n* = 18).

^†^ Testing only performed in subset of children who were eligible for sputum induction (*n* = 236). Data not available for some children: HIV (*n* = 6, due to parent refusal).

### Parasitological outcomes.

**Figure 1.**
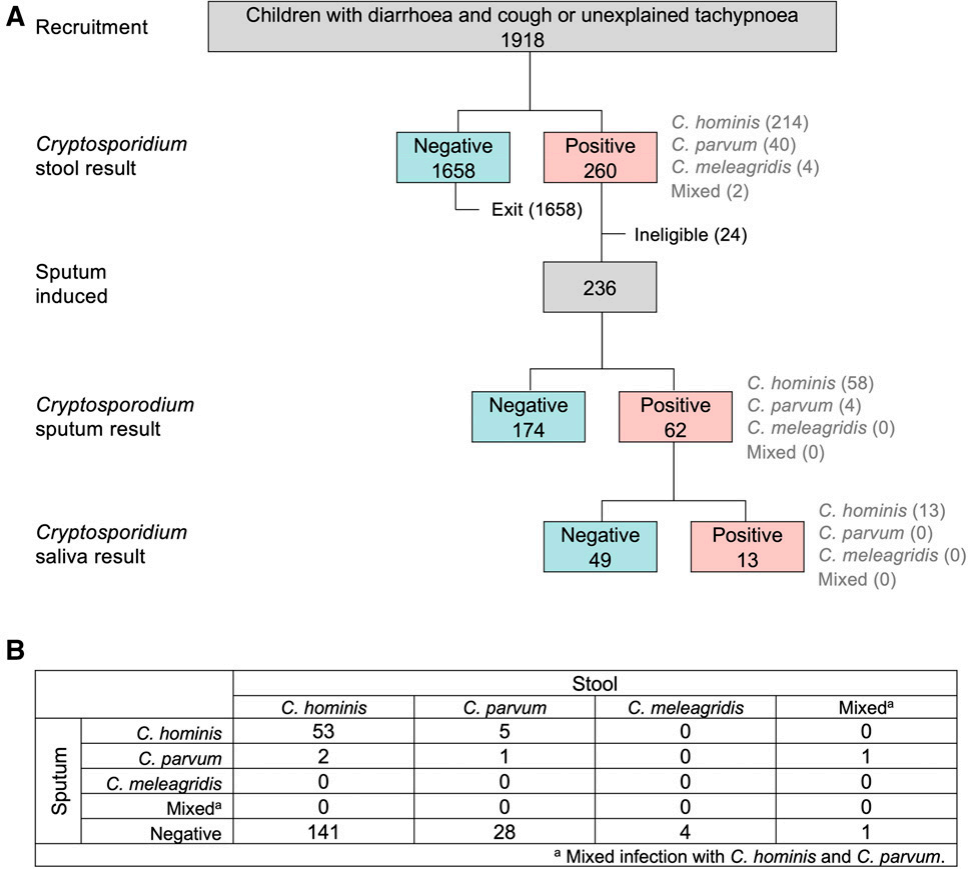
*Cryptosporidium* status and species of children aged 9 to 36 months presenting with diarrhea and cough or unexplained tachypnea to the Acute Care Unit of Mulago Hospital, Kampala, Uganda (*N* = 1,918). (**A**) Flow chart depicting *Cryptosporidium* status and species by sample type (red = positive, aqua = negative). (**B**) Species of *Cryptosporodium* detected in stool and sputum of subset of children who underwent sputum induction (*n* = 236).

Results of parasitological testing are shown in Figure 1. Overall, 260 (13.6%) children tested positive for *Cryptosporidium* in the stool. *C. hominis* was associated with more than 80% of stool-positive cases. Of 236 children who had sputum available for testing, 62 children (26.3%) had *Cryptosporidium* in the sputum, 13 (5.5% overall; 21.0% of 62) of whom also had *Cryptosporidium* in the saliva. Respiratory involvement was more common in children with *C. hominis* in the stool, although this was not statistically significant (56/198 = 28.3% of *C. hominis* intestinal infections versus 7/36 = 19.4% of *C. parvum* intestinal infections; *P* = 0.271). Discordancy between *Cryptosporidium* species present in stool and sputum was noted in some children. This included two children who had *C. hominis* and *C. parvum* in the stool and sputum, respectively; five children who had *C. parvum* and *C. hominis* in the stool and sputum, respectively; and one child who had mixed (*C. hominis* and *C. parvum*) in the stool but only *C. parvum* in the sputum.

### Clinical outcomes by *Cryptosporidium* status.

**Figure 2.**
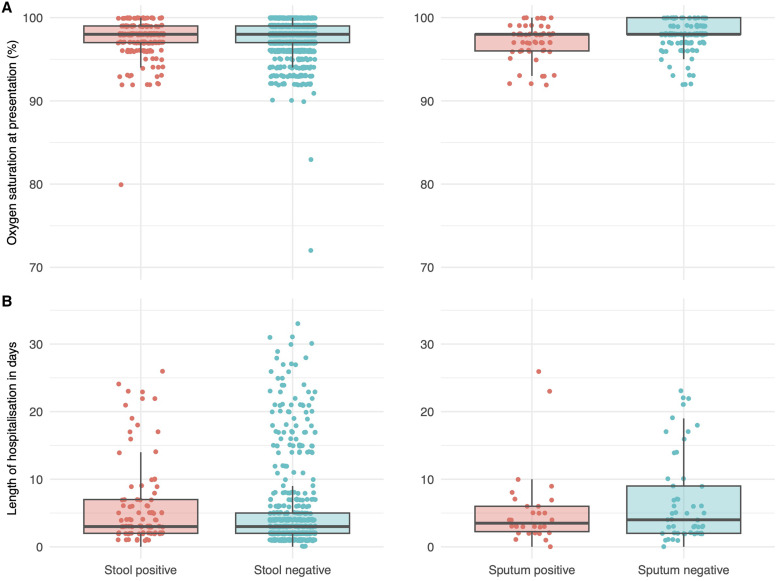
(**A**) Oxygen saturation at presentation and (**B**) length of hospitalization of children aged 9 to 36 months presenting with diarrhea and cough or unexplained tachypnea to the Acute Care Unit of Mulago Hospital, Kampala, Uganda, by *Cryptosporidium* status (red = positive, aqua = negative). Panels on left show children stratified by stool status (*N* = 1,918), and panels on right show children stratified by sputum status (*n* = 236). Individual patient values are indicated as dots with the median and interquartile range indicated as a boxplot.

Clinical signs and symptoms, concurrent respiratory infections, and clinical end point of children are shown in Table 2 and Figure 2. Children with intestinal cryptosporidiosis had a longer duration of diarrhea (*P* <0.001) and were more likely to have a history of vomiting (*P* <0.001) at presentation compared with stool-negative children. There was no clear association between intestinal *Cryptosporidium* infection and presence or severity of respiratory signs and symptoms, with the possible exception of lower frequency of tachypnea and higher frequency of chest indrawing in stool-positive children (*P* = 0.024 and 0.05, respectively). However, this association was not apparent when analysis was limited to children with and without confirmed respiratory *Cryptosporidium* infection. Oxygen saturations were slightly but significantly lower in the children with positive sputum (*P* <0.001), with such children more likely to have abnormal saturations (SpO_2_ < 96%; *P* = 0.053). Although most children had concurrent bacterial infections, the proportion of children with positive bacterial culture was lower in children with respiratory *Cryptosporidium* infection compared with children who did not have *Cryptosporidium* infection at this site (*P* = 0.044). Stool-positive children experienced longer length of hospitalization (*P* = 0.005) and were more likely to die (*P* <0.001) compared with children without intestinal infection. Children with respiratory *Cryptosporidium* infection were more likely to be hospitalized (*P* = 0.010) compared with children who did not have the respiratory infection.

**Table 2:** Clinical signs and symptoms, concurrent respiratory infections, and clinical end point of children aged 9 to 36 months presenting with diarrhea and cough or unexplained tachypnea to the Acute Care Unit of Mulago Hospital, Kampala, Uganda, by *Cryptosporidium* status (*N* = 1,918).

Clinical Characteristic	All Children (*N* = 1,918)			Stool-Positive Children Who Underwent Sputum Induction (*n =* 236)		
	Stool Result		*P*	Sputum Result		*P*-Value
	Positive (*n* = 260)	Negative (*n* = 1,658)		Positive (*n* = 62)	Negative (*n* = 174)	
Gastrointestinal signs and symptoms						
Diarrhea	260 (100)	1,658 (100)	NA	62 (100)	174 (100)	NA
Duration of diarrhea in days, median (IQR)*	7 (8)	4 (5)	<0.001	7 (10)	7 (7)	0.017
History of persistent diarrhea*	22 (8.5)	158 (9.5)	0.648	5 (8.1)	14 (8)	1
History of recurrent diarrhea*	120 (46.2)	806 (48.6)	0.464	20 (32.3)	90 (51.7)	0.011
Recent history of vomiting	189 (72.7)	1,015 (61.2)	<0.001	46 (74.2)	125 (71.8)	0.869
Respiratory signs and symptoms						
Cough	260 (100)	1,651 (99.6)	0.603	62 (100)	174 (100)	1
Duration of cough in days, median (IQR)*	7 (4)	7 (4)	0.892	5.5 (11)	7 (4)	0.889
History of difficulty breathing	78 (30)	553 (33.4)	0.320	19 (30.6)	51 (29.3)	0.872
History of wheezing	10 (3.8)	69 (4.2)	1	4 (6.5)	4 (2.3)	0.212
Tachypnea*	15 (5.8)	171 (10.3)	0.024	2 (3.2)	12 (6.9)	0.366
Oxygen saturation at presentation, median (IQR)*	98 (2)	98 (2)	0.865	98 (2)	98 (2)	<0.001^†^
Oxygen saturation <96% at presentation*	22 (8.5)	102 (6.2)	0.174	9 (14.5)	10 (5.7)	0.053
Hypoxia (SpO_2_ <92%)*	1 (0.4)	6 (0.4)	1	0	0	NA
Cyanosis	0	1 (0.1)	1	0	0	NA
Crepitations	1 (0.4)	17 (1)	0.496	1 (1.6)	0	0.263
Lower chest indrawing	4 (1.5)	7 (0.4)	0.050	0	3 (1.7)	0.569
Bronchial breathing	3 (1.2)	4 (0.2)	0.057	2 (3.2)	1 (0.6)	0.170
Wheezing	0 (0)	2 (0.1)	1	0	0	NA
Rhonchi	0 (0)	4 (0.2)	1	0	0	NA
Concurrent respiratory infections						
Positive bacterial culture^‡^	NA	NA	NA	38 (62.3)^§^	133 (76.4)^ǁ^	0.044
Positive TB smear^‡^	NA	NA	NA	2 (3.3)	3 (1.7)	0.607
Positive PJP smear^‡^	NA	NA	NA	1 (1.9)	9 (5.7)	0.457
Clinical end point						
Admitted to hospital	100 (38.5)	578 (34.9)	0.265	32 (51.6)	57 (32.8)	0.010
Length of hospitalization in days, median (IQR)^†^	3 (5)	3 (3)	0.005^¶^	3 (4.5)	4 (7)	0.637
Death	5 (1.9)	1 (0.1)	<0.001	1 (1.6)	1 (0.6)	0.457

IQR = interquartile range; NA = not applicable; PJP = *Pneumocystis jirovecii* pneumonia; TB = tuberculosis. Values are *n* (%) unless otherwise stated.

All children were tested for *Cryptosporidium* in the stool (*N* = 1,918). A subset of stool-positive children underwent sputum induction and were also tested for *Cryptosporidium* in the sputum (*n* = 236).

* Data not available for some children: diarrhea duration (*n* = 1), persistent diarrhea (*n* = 1), recurrent diarrhea (*n* = 1), cough duration (*n* = 6), tachypnea (*n* = 2), O_2_ saturation/hypoxia (*n* = 1), and hospitalization length (*n* = 18).

^†^ Note that the data are not normally distributed (see Figure 2); the variance is different between groups despite having the same median and IQR (*P* <0.001 on Mann–Whitney *U* test).

^‡^ Testing only performed in subset of children who were eligible for sputum induction. Data not available for some children: microbiology (*n* = 1) and TB result (*n* = 1); PJP (26).

^§^ Bacterial pathogens present in sputum: *Streptococcus pneumoniae* (*n* = 9), *Klebsiella pneumoniae* (*n* = 11), *Escherichia coli* (*n* = 2), mixed (*n* = 9), and other pathogen (*n* = 7).

^ǁ^ Bacterial pathogens present in sputum: *Streptococcus pneumoniae* (*n* = 27), *Klebsiella pneumoniae* (*n* = 21), *Escherichia coli* (*n* = 21), mixed (*n* = 38), and other pathogen (*n* = 26).

^¶^ Note that the data are not normally distributed (see Figure 2); the variance is different between groups despite having the same median (*P* = 0.005 on Mann–Whitney *U* test).

### Factors associated with respiratory cryptosporidiosis and hospitalization.

Table 3 shows the association between *Cryptosporidium* sputum status and potential risk factors in stool-positive children who underwent sputum testing. None of the variables examined (gender, age, HIV status, nutritional status, history of vomiting, presence of *C. hominis* in the stool) were associated with respiratory infection. Table 4 shows the association between hospitalization and potential risk factors. After adjusting for other factors (gender, age, HIV status, nutritional status, hydration status), children with *Cryptosporidium* in their sputum had 2.1 times the odds of hospitalization compared with children without *Cryptosporidium* in their sputum (adjusted odds ratio = 2.08, 95% CI: 1.02–4.22; *P* = 0.043).

**Table 3:** Factors associated with respiratory cryptosporidiosis in stool-positive children aged 9 to 36 months who presented with diarrhea and cough or unexplained tachypnea and underwent sputum induction at the Acute Care Unit of Mulago Hospital, Kampala, Uganda (*n* = 236)

Independent Variables	Sputum Result, *n* (%)		cOR (95% CI)	*P*-Value	aOR (95% CI)	*P*-Value
	Positive	Negative				
History of vomiting						
No	16 (24.6)	49 (75.4)	1		1	
Yes	46 (26.9)	125 (73.1)	1.13 (0.58–2.18)	0.722	1.04 (0.53–2.07)	0.903
*Cryptosporidium hominis* in stool						
No	6 (15.8)	32 (84.2)	1		1	
Yes	56 (28.3)	142 (71.7)	2.10 (0.83–5.3)	0.155	2.16 (0.84–5.55)	0.109
Gender						
Male	37 (27.6)	97 (72.4)	1		1	
Female	25 (24.5)	77 (75.5)	0.85 (0.47–1.513)	0.592	0.82 (0.44–1.53)	0.535
Age, months						
9–12	31 (25.8)	89 (74.2)	1		1	
13–24	29 (27.6)	76 (72.4)	1.10 (0.61–2.0)	0.763	1.12 (0.63–2.28)	0.582
25–36	2 (18.2)	9 (81.8)	0.64 (0.13–3.1)	0.579	1.01 (0.19–5.41)	0.990
HIV status						
Negative	60 (26.8)	164 (73.2)	1		1	
Positive	2 (33.3)	4 (66.7)	1.37 (0.24–7.66)	0.772	2.41 (0.37–15.79)	0.358
Nutritional status						
Not underweight	39 (24.5)	120 (75.5)	1		1	
Underweight (WAZ <−2.0)	23 (30.3)	53 (69.7)	1.34 (0.73–2.45)	0.352	1.13 (0.43–2.95)	0.809
Not stunted	52 (28.0)	134 (72.0)	1		1	
Stunted (HAZ <−2.0)	10 (20.8)	38 (79.2)	0.68 (0.31–1.46)	0.321	0.50 (0.19–1.30)	0.154
Not wasted	37 (23.6)	120 (76.4)	1		1	
Wasted (WHZ <−2.0)	22 (30.6)	50 (69.4)	1.43 (0.77–2.66)	0.263	1.39 (0.58–3.31)	0.463

aOR = adjusted odds ratio; cOR = crude odds ratio; HAZ = height-for-age *Z* score; WAZ = weight-for-age *Z* score; WHZ = weight-for-height *Z* score.

**Table 4:** Factors associated with hospitalization in stool-positive children aged 9 to 36 months who presented with diarrhea and cough or unexplained tachypnea and underwent sputum induction at the Acute Care Unit of Mulago Hospital, Kampala, Uganda (*n* = 236)

Independent Variables	Hospitalized, *n* (%)		cOR (95% CI)	*P*-Value	aOR (95% CI)	*P*-Value
	Yes	No				
*Cryptosporidium* in sputum						
No	57 (32.8)	117 (67.2)	1		1	
Yes	32 (51.6)	30 (48.4)	2.19 (1.21–3.95)	0.009	2.08 (1.02–4.22)	0.043
Gender						
Male	50 (37.3)	84 (62.7)	1		1	
Female	39 (38.2)	63 (61.8)	1.04 (0.61–1.77)	0.885	0.99 (0.51–1.99)	0.970
Age, months						
9–12	45 (37.5)	75 (62.5)	1		1	
13–24	43 (41.0)	62 (59.0)	1.16 (0.68–1.98)	0.597	0.90 (0.56–1.78)	0.765
25–36	1 (9.1)	10 (90.9)	0.17 (0.02–1.35)	0.093	0.26 (0.03–2.54)	0.246
HIV status						
Negative	83 (37.1)	141 (62.9)	1		1	
Positive	5 (83.1)	1 (16.7)	8.49 (0.98–74.0)	0.053	8.01 (0.71–89.37)	0.091
Nutritional status						
Not underweight	46 (28.9)	113 (71.1)	1		1	
Underweight (WAZ <−2.0)	42 (55.3)	34 (44.7)	3.04 (1.72–5.35)	<0.001	1.66 (0.62–4.43)	0.311
Not stunted	68 (36.6)	118 (63.4)	1		1	
Stunted (HAZ <−2.0)	21 (43.8)	27 (56.3)	1.35 (0.71–2.57)	0.361	1.05 (0.40–2.71)	0.928
Not wasted	43 (27.4)	114 (72.6)	1		1	
Wasted (WHZ <−2.0)	42 (58.3)	30 (41.7)	3.71 (2.07–6.66)	<0.001	2.26 (0.95–5.39)	0.067
Hydration status						
No dehydration	55 (28.2)	140 (71.8)	1		1	
Some/severe dehydration	34 (82.9)	7 (17.1)	12.4 (5.17–29.55)	<0.001	10.44 (4.12–26.47)	<0.001
Concurrent bacterial infection						
No	24 (37.5)	40 (62.5)	1		1	
Yes	65 (38.0)	106 (62.0)	1.02 (0.57–1.85)	0.943	0.77 (0.37–1.60)	0.485

aOR = adjusted odds ratio; cOR = crude odds ratio; HAZ = height-for-age *Z* score; WAZ = weight-for-age *Z* score; WHZ = weight-for-height *Z* score.

## DISCUSSION

This study confirms that respiratory cryptosporidiosis is common in stool-positive children with diarrhea and respiratory symptoms. It was not associated with HIV infection nor malnutrition in this patient population. Although the frequency of tachypnea at presentation was similar between children with and without respiratory infection, oxygen saturations were lower in children who underwent sputum induction with respiratory cryptosporidiosis. Stool-positive children who had *Cryptosporidium* in the sputum were more likely to be hospitalized than stool-positive children who were sputum-negative. This finding persisted after adjusting for HIV status, malnutrition, dehydration, and concurrent respiratory bacterial infection. Because decisions to hospitalize were made independent of *Cryptosporidium* status, these findings suggest that sputum-positive children had more serious illness than their sputum-negative counterparts. We conjecture that in children with respiratory symptoms, it may be particularly prudent to use agents such as nitazoxanide that are systemically absorbed. Antiparasitic agents that act in the lumen of the intestine but are not absorbed, such as paromomycin, cannot be expected to treat infection in this site.

*Cryptosporidium* remains an important cause of illness in children presenting to ACUs in Uganda. The prevalence of intestinal cryptosporidiosis in this study (13% of children with diarrhea and cough using PCR) was about the same as in our pilot study (12.5% of children with diarrhea and cough using PCR^19^) but lower than our earlier studies, which focused exclusively on diarrhea (25% of children with diarrhea using modified acid-fast stain^27^; 31% children with persistent diarrhea using modified acid-fast stain^26^). These latter studies were undertaken at a time when HIV infection was more common, malnutrition more prevalent, and molecular methods to discriminate between *C. hominis* and *C. parvum* were not used, likely contributing to the higher rates of diagnosis. Nevertheless, consistent with these previous studies, we found that stool positivity for *Cryptosporidium* was associated with longer duration of diarrhea, higher rates of malnutrition (data not shown), and more frequent death than in children with diarrhea due to other causes. To that end, we emphasize that overall, *Cryptosporidium* is a highly important and clinically significant infection in children in low-income countries, including in Uganda.

Few studies have systematically investigated respiratory cryptosporidiosis in pediatric patients. The high frequency of respiratory involvement in stool-positive children with diarrhea and respiratory symptoms in this study (26%) is similar to findings from our pilot study (17/48 or 35%^19^). It is also consistent with findings from a recent study of Malawian children experiencing diarrhea, where approximately two-thirds of patients exhibited cough symptoms (11/37 or 31% of children at the baseline visit^18^). Although common, we cannot comment on whether respiratory infection is transient or precedes or follows intestinal infection given the cross-sectional design of our study. However, in Malawi, repeated sampling of children over 8 weeks revealed that respiratory infection persisted over several weeks and was associated with longer (albeit statistically nonsignificant) gastrointestinal shedding.^18^ This suggests that further studies investigating the natural evolution of cryptosporidiosis in patients over time may show a higher cumulative respiratory involvement rate.

The precise mechanism through which the protozoan enters the respiratory tract remains unknown. Aspiration from the gastrointestinal tract during vomiting is plausible and supported by the detection of *Cryptosporidium* in saliva of some children in this and our previous study,^19^ as well as in the nasopharynx of some children in the Malawi study.^18^ We previously showed that adults with suspected TB expectorate *Cryptosporidium* in the sputum suggesting the potential for dissemination or transmission via this route.^17^ Hence it is also possible that children acquire the infection directly through the respiratory tract. Subsequent replication in this site, coupled with coughing and subsequent ingestion of sputum, could result in gastrointestinal infection. Although not proven, we speculate that respiratory infection may play a role in mother-to-child transmission of anthroponotic *Cryptosporidium* through the close contact afforded by this relationship. Further studies are needed to confirm or refute these hypotheses.

We found some evidence to suggest that cryptosporidiosis was associated with respiratory compromise. Children with confirmed respiratory cryptosporidiosis had lower oxygen saturations at presentation than other children who underwent sputum induction, although this difference was not apparent in children with and without *Cryptosporidium* in the stool. In the Malawi study, respiratory rates, oxygen saturation and frequencies of respiratory symptoms were similar between stool-positive and stool-negative children.^18^ We focused our study on patients with cough or unexplained tachypnea to enrich the sample with children who would be eligible for sputum induction according to our approved ethics protocol. As such, we cannot assess differences in the frequency of cough between groups. Previous studies have, however, found that children with *Cryptosporidium* diarrhea are more likely to have cough than those with diarrhea due to other causes.^25^ We speculate that *Cryptosporidium* was the likely cause of respiratory signs and symptoms in at least some of the children in our study, as evidenced by the fact that nearly 40% of sputum-positive children in our study had negative bacterial cultures (compared with only 24% of sputum-negative children, *P* = 0.044). No studies have systematically investigated respiratory viral pathogens in our patient population however analysis of a small number of samples from this study (*n* = 48) reveals that viral carriage is high (>90%, unpublished). The clinical significance of this finding is not clear and warrants further investigation.

We characterized children using parameters (gender, age, HIV status, nutritional and hydration status, concurrent bacterial infection) that have been proven to predict adverse outcomes, including hospitalization, in many other circumstances. When adjusted for these, however, the risk of hospitalization was still significantly higher for children with *Cryptosporidium* in sputum compared with those with only enteric infection. This implies that the presence of *Cryptosporidium* in the respiratory tract represents a severe presentation. If confirmed, then respiratory cryptosporidiosis may be a useful prognostic marker. We speculate that respiratory involvement above and beyond enteric involvement may indicate enhanced susceptibility and/or greater parasite burden. Numerous historical case reports have emphasized respiratory cryptosporidiosis as a feature of disseminated disease in immunocompromised patients.^20^ However, it is also plausible that in this age group, this reflects the first time that some children have been infected and thus they have no prior immunity. This could result in higher parasite burden. The latter is supported by a study in Malawi, which found that children with respiratory cryptosporidiosis tended to shed the parasite for a longer period than those whose infection was limited to the gastrointestinal tract.^18^

This study has several limitations that should be noted. First, our focus on children who were eligible for sputum induction may have introduced selection bias through the exclusion of children with mild or severe respiratory symptoms. Second, in our original protocol, we outlined a plan to recruit 2,894 children, projecting an enrollment of approximately 60 children per month over a 48-month period, with an anticipated 25% of children being *Cryptosporidium* stool positive. However, we encountered several challenges that necessitated deviations from this protocol. This included lower-than-expected recruitment rates (averaging 40 children per month), and a lower-than-anticipated stool-positivity rate (13%). In addition, initial attempts to use immunofluorescence for stool screening resulted in some misdiagnoses, prompting us to introduce PCR. Consequently, data from the early recruitment period (before introduction of PCR) was excluded from analysis. These factors combined mean that our study may not have had sufficient power to detect differences between groups in terms of outcomes such as tachypnea and oxygen saturation at presentation.

## CONCLUSION

Respiratory tract involvement is common in children with intestinal cryptosporidiosis who are experiencing respiratory symptoms. This finding is not modulated by HIV infection or malnutrition. Children with respiratory tract involvement may experience some degree of respiratory compromise and may be at increased risk for hospitalization. Future efforts to develop therapeutics for cryptosporidiosis should ensure that drugs have activities against multiple mucosal surfaces, including the respiratory tract, to enhance treatment efficacy.
